# Acute myeloid leukaemia niche regulates response to L‐asparaginase

**DOI:** 10.1111/bjh.15924

**Published:** 2019-04-24

**Authors:** Gertjan J. L. Kaspers

**Affiliations:** ^1^ Princess Máxima Center for Paediatric Oncology Amsterdam UMC Vrije Universiteit Amsterdam Amsterdam The Netherlands

**Keywords:** AML: heterogeneity, ASNS expression, cathepsin‐B expression

L‐asparaginase is an enzyme that causes hydrolysis of L‐asparagine and L‐glutamine, leading to its reduction and, ultimately, depletion in peripheral blood and bone marrow. If cells cannot produce these essential amino acids themselves, they will die upon exposure to L‐asparaginase. This drug is particularly effective in lymphoblastic leukaemias and lymphomas, because these cells typically lack the enzyme asparagine synthetase (ASNS), required for the formation of asparagine, and because these cells are susceptible to asparagine depletion. In contrast, acute myeloid leukaemia (AML) cells have variable expression of ASNS, and seem particularly susceptible to glutamine depletion. This explains why L‐asparaginase is not commonly used as anti‐leukaemic agent in AML regimen. However, factors other than intracellular ASNS activity also play a role in resistance and sensitivity to L‐asparaginase. For instance, L‐asparaginase may be inactivated by the lysosomal cysteine protease cathepsin B (CTSB) and by asparaginyl endopeptidase. In addition, it is now well known that L‐asparaginase may be inactivated by neutralizing antibodies, associated with or even without (silent inactivation) signs and symptoms in patients.

In this issue of the journal, Michelozzi *et al* (2019) report that the leukaemic stem cells in AML samples, both the CD34+/CD38+and the CD34+/CD38− fractions, are intrinsically sensitive to L‐asparaginase. However, cells in the bone marrow microenvironment seem to produce CTSB, which inactivates L‐asparaginase. These cells include mesenchymal stromal cells and monocytes/macrophages, and they may also produce other factors as well, potentially also interfering with the effect of L‐asparaginase. Of interest, AML cells itself may also produce CTSB; all of this contributes to increased resistance to L‐asparaginase. The authors conclude that L‐asparaginase might have anti‐leukaemic activity in AML cells in general, and in leukaemic stem cell populations in particular, if the protective effect of the bone marrow microenvironment can be overcome.

In agreement with this study, the literature shows that, while L‐asparaginase was effective in individual patients with AML (Ohnuma *et al*, 1969; Hansen & Canellos, 1970), and also had efficacy when combined with methotrexate in paediatric refractory/relapsed AML (Buaboonnam *et al*, 2013) or with high‐dose cytarabine in both adults and children (Capizzi *et al*, 1988; Wells *et al*, 1993), it is generally much less useful in AML than in ALL (Keating *et al*, 1993). Extensive *in vitro* drug resistance testing by our laboratory of childhood acute leukaemia samples in cell suspensions without stroma support, also revealed that AML cells are, on average, 7‐fold more resistant to L‐asparaginase than ALL cells (Zwaan *et al*, 2000). The protective effect of bone marrow stroma on drug‐induced leukaemic cell kill has also been reported by others, for antileukaemic agents other than L‐asparaginase, such as cytarabine (Konopleva *et al*, 2002; Matsunaga *et al*, 2003).

So, which are the perspectives for L‐asparaginase in AML? First, we should consider the higher susceptibility of AML cells to glutamine depletion. Of interest, Erwinia L‐asparaginase (Erwinase) has a 10‐fold higher glutaminase activity than E. Coli L‐asparaginase. Erwinase lead to glutamine depletion in plasma and had anti‐leukaemic activity in 2 out of 5 AML patients as a single agent (Emadi *et al*, 2018). Willems *et al* (2013) reported that AML cells are susceptible to L‐asparaginase‐induced glutamine depletion, causing downregulating of the mTORC1 signalling pathway and resulting in apoptosis of AML cells. Thus, Erwinase seems the better alternative to test further in AML. An alternative could be an L‐asparaginase analogue that is less prone to proteolytic cleavage, while maintaining its enzymatic activity.

Second, in order to learn more about interindividual differences in the efficacy of L‐asparaginase in general, or Erwinase in particular, in AML, window studies with single‐agent Erwinase seem appropriate. Such an approach also facilitates associated biological studies, such as on the influence of factors produced by patient‐specific bone marrow stromal cells, and the AML cells themselves. Obvious candidates are ASNS and CTSB, as well as asparagine and glutamine. At the same time, it is now technically feasible to study not only the disappearance, if any, of the bulk of AML cells, but also of specific subpopulations, as defined by multiparameter flowcytometry (Cloos *et al*, 2018). Of course, AML is a heterogeneous disease, and detailed clinical studies may reveal whether subgroups, as defined by cytogenetic features, are more or less sensitive to L‐asparaginase. Indeed, AML with 7q‐ or monosomy 7 might be more sensitive, because these AML cells seem to produce less ASNS (Bertuccio *et al*, 2017). Moreover, AML with *IDH1* or *IDH2* mutations may be particularly sensitive to glutamine depletion, as reported by Fathi *et al* (2015).

Third, L‐asparaginase should be tested in combination with other drugs that counteract any factor resulting in its decreased efficacy. Obvious candidates are inhibitors of CTSB and/or ASNS, which may be possible with specific protease inhibitors. Interestingly, cytarabine may down‐regulate *ASNS* transcription and thus has the potential of synergy with L‐asparaginase. Indeed, Capizzi *et al* (1988) reported on a randomized study that showed that the addition of L‐asparaginase to high‐dose cytarabine lead to a significantly improved complete remission rate and overall survival benefit. It is important to emphasize that the sequence of both drugs is important, with L‐asparaginase to follow cytarabine. Similarly, in a large clinical study in paediatric AML, Wells *et al* (1993) demonstrated good anti‐leukaemic effect of cytarabine plus L‐asparaginase (CLASP), although this was schedule‐dependent, with better efficacy if CLASP was given every 7 days as compared to every 28 days. Another approach to counteract the protective effect of the bone marrow stroma on residual AML cells, is to interfere with the binding of these residual cells to the stroma by agents such as plerixafor, an CXCR4 antagonist (Martínez‐Cuadrón *et al*, 2018).

In general, the schedule of L‐asparaginase must also be considered. From studies in ALL we have learned that prolonged depletion of asparagine is most relevant for an optimal anti‐leukaemic effect. The same is likely to be true for depletion of glutamine. Thus, repeated and uninterrupted administrations of L‐asparaginase is likely to be optimal. The latter is also important to avoid the development of neutralizing antibodies. Finally, therapeutic drug monitoring should be introduced. Important interindividual differences in systemic exposure after a given dose of L‐asparaginase have been observed. By simply measuring the asparaginase activity levels in peripheral blood, individualized drug dosing Is possible. This minimizes costs and avoids unnecessary toxicity, and at the same time optimizes efficacy (van der Sluis *et al*, 2016).

In conclusion, the study reported by Michelozzi *et al* (2019) in this issue of the British Journal of Haematology should encourage further studies on the potential beneficial effect of L‐asparaginase in AML, and how to optimize that benefit.
